# Displacement of Hem-o-lok clips into the common bile duct after LCBDE: a case report

**DOI:** 10.3389/fsurg.2026.1806110

**Published:** 2026-03-20

**Authors:** Huan Wu, Xin Li, Liang Zhang

**Affiliations:** 1 Department of Anesthesiology, The Operating Room of Pengzhou Second People’s Hospital, Chengdu, Sichuan Province, China; 2 Department of General Surgery, Second People’s Hospital of Pengzhou City, Chengdu, Sichuan Province, China

**Keywords:** bile duct, case report, common bile duct stones, Hem-o-lok clip migration, laparoscopic cholecystectomy

## Abstract

**Background:**

Laparoscopic cholecystectomy (LC) combined with laparoscopic common bile duct exploration (LCBDE) is the standard treatment for hepatobiliary calculi. While postoperative complications have decreased with technical maturation, migration of polymer ligation clips (e.g., Hem-o-lok) remains an underrecognized yet serious complication that can serve as a nidus for recurrent stone formation and cholangitis, necessitating heightened clinical awareness.

**Methods:**

We present a case of a 76-year-old female who presented with fever and chills three years after initial LCBDE. Imaging studies (CT/MRI) revealed choledocholithiasis with a central linear hyperdensity suggestive of migrated clips. The patient underwent laparoscopic bile duct re-exploration.

**Results:**

Intraoperative findings confirmed multiple Hem-o-lok clips within the common bile duct, one embedded at the core of a large stone. All clips and stones were successfully removed, followed by T-tube drainage. The patient recovered rapidly with targeted antibiotic therapy and was discharged on postoperative day three.

**Conclusion:**

Although rare, clip migration should be considered in patients with late-onset biliary symptoms after LCBDE. This case underscores the importance of meticulous clip application and suggests the need for long-term follow-up and possibly alternative closure techniques to prevent this consequential complication.

## Introduction

Laparoscopic cholecystectomy (LC) has firmly established itself as the gold standard for the management of symptomatic cholelithiasis and benign gallbladder diseases, owing to its well-documented advantages of reduced postoperative pain, shorter hospital stay, and superior cosmesis compared to open surgery (1). With advancing techniques and instrumentation, the scope of minimally invasive biliary surgery has expanded to include laparoscopic common bile duct exploration (LCBDE) for concomitant choledocholithiasis, offering a single-stage, definitive treatment solution (2).

Despite its widespread success and low overall morbidity, LC and LCBDE are not devoid of complications. Beyond the immediate perioperative risks such as bleeding or bile leak, a spectrum of delayed complications can occur, including post-cholecystectomy syndrome, bile duct strictures, and retained or recurrent common bile duct stones (3, 4). Among these, the migration of non-absorbable polymer ligation clips (e.g., Hem-o-lok) into the biliary tree is a particularly rare and insidious complication that remains underrecognized in clinical practice (5, 6). The displaced clip can act as a nidus for the formation of secondary calculi, leading to obstructive jaundice, recurrent cholangitis, or pancreatitis, often manifesting years after the initial surgery (7, 8).

The diagnosis of clip migration poses a significant challenge. Its clinical presentation—fever, abdominal pain, and jaundice—is nonspecific and overlaps with more common conditions like *de novo* choledocholithiasis (6, 9). While imaging modalities such as computed tomography (CT) or magnetic resonance cholangiopancreatography (MRCP) may reveal tell-tale linear hyperdensities within the bile duct, these findings are frequently misinterpreted as primary stones or overlooked altogether (10, 11). Furthermore, the underlying mechanisms facilitating clip dislocation—whether technical factors during application, local inflammatory processes, or the formation of a T-tube sinus tract—are not fully elucidated and are primarily inferred from isolated case reports (12, 13). Currently, the literature on Hem-o-lok clip migration consists predominantly of individual case reports or small series, with a limited synthesis of its clinical trajectory, optimal management strategies, and preventive measures (6, 8, 14, 15). There is a need to consolidate this scattered evidence to heighten clinical vigilance and guide decision-making.

Therefore, we present a detailed case of a patient who developed recurrent cholangitis and obstructive jaundice due to Hem-o-lok clip migration into the common bile duct three years after LCBDE. Through this report and an accompanying review of pertinent literature, we aim to: (1) underscore the importance of considering clip migration in the differential diagnosis of late biliary complications post-LCBDE, (2) discuss the diagnostic clues and therapeutic approaches based on accumulated experience, and (3) hypothesize on potential etiological factors to inform preventative surgical technique.

## Case presentation

A 76-year-old female presented to the emergency department with a two-day history of acute-onset fever and rigors, accompanied by anorexia and right upper quadrant discomfort. Her medical history was significant for a prior laparoscopic cholecystectomy (LC) with common bile duct exploration (LCBDE) and T-tube drainage, performed at our institution four years earlier for choledocholithiasis and acute suppurative cholangitis. On examination, she was febrile (38.3 °C) with localized tenderness and mild guarding in the right upper abdomen. Laboratory studies revealed a cholestatic and hepatitic pattern: total bilirubin 45.4 µmol/L, direct bilirubin 31.3 µmol/L, alanine aminotransferase 156 U/L, aspartate aminotransferase 251 U/L, and a markedly elevated procalcitonin of 16.95 ng/mL, indicative of a severe bacterial infection.

**Figure 1:**
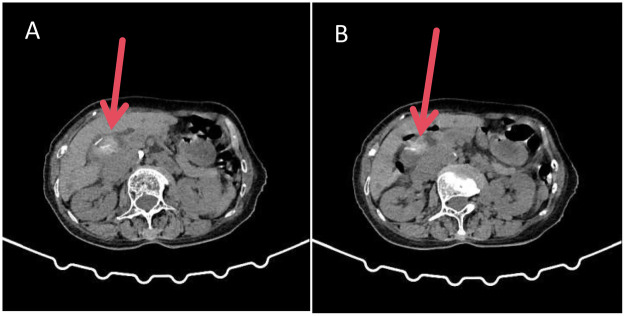
Preoperative upper abdominal CT images. **(A)** Axial view showing a dilated common bile duct with multiple hyperdense nodules. **(B)** Coronal view revealing a distinct linear hyperdensity at the center of a dominant stone in the proximal common bile duct (arrow), suggestive of a migrated clip.

**Figure 2:**
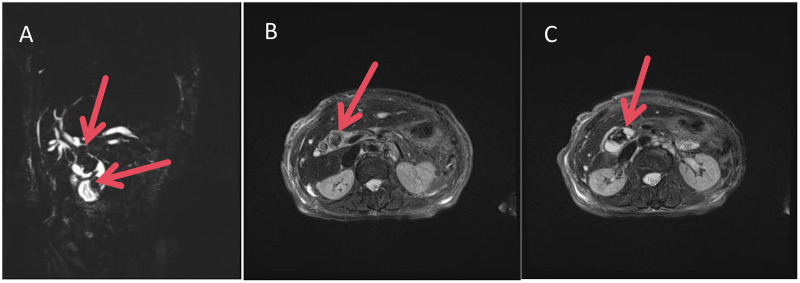
Preoperative upper abdominal MRI. **(A)** MRCP image demonstrating diffuse dilatation of the intra- and extrahepatic biliary tree with multiple filling defects. **(B)** Axial T2-weighted image confirming nodular filling defects in the common bile duct. **(C)** Another axial T2-weighted image (different slice) showing additional filling defects with red arrows indicating areas of abnormality in the biliary tree.

Abdominal ultrasonography confirmed the absence of the gallbladder and identified a dilated common bile duct (13 mm) containing hyperechoic material. Subsequent contrast-enhanced computed tomography (CT) of the abdomen was pivotal. It demonstrated diffuse dilatation of the intra- and extrahepatic biliary tree with multiple hyperdense nodules consistent with stones. Critically, a distinct linear hyperdensity was visualized at the center of a dominant stone within the proximal common bile duct (Figures 1A,B), raising the first suspicion of a migrated foreign body as the nidus. Magnetic resonance imaging (MRI) further corroborated these findings, revealing multiple nodular filling defects throughout the biliary system (Figure 2). The collective findings established the diagnosis of recurrent common bile duct stones with associated acute suppurative cholangitis and obstructive jaundice.

**Figure 3:**
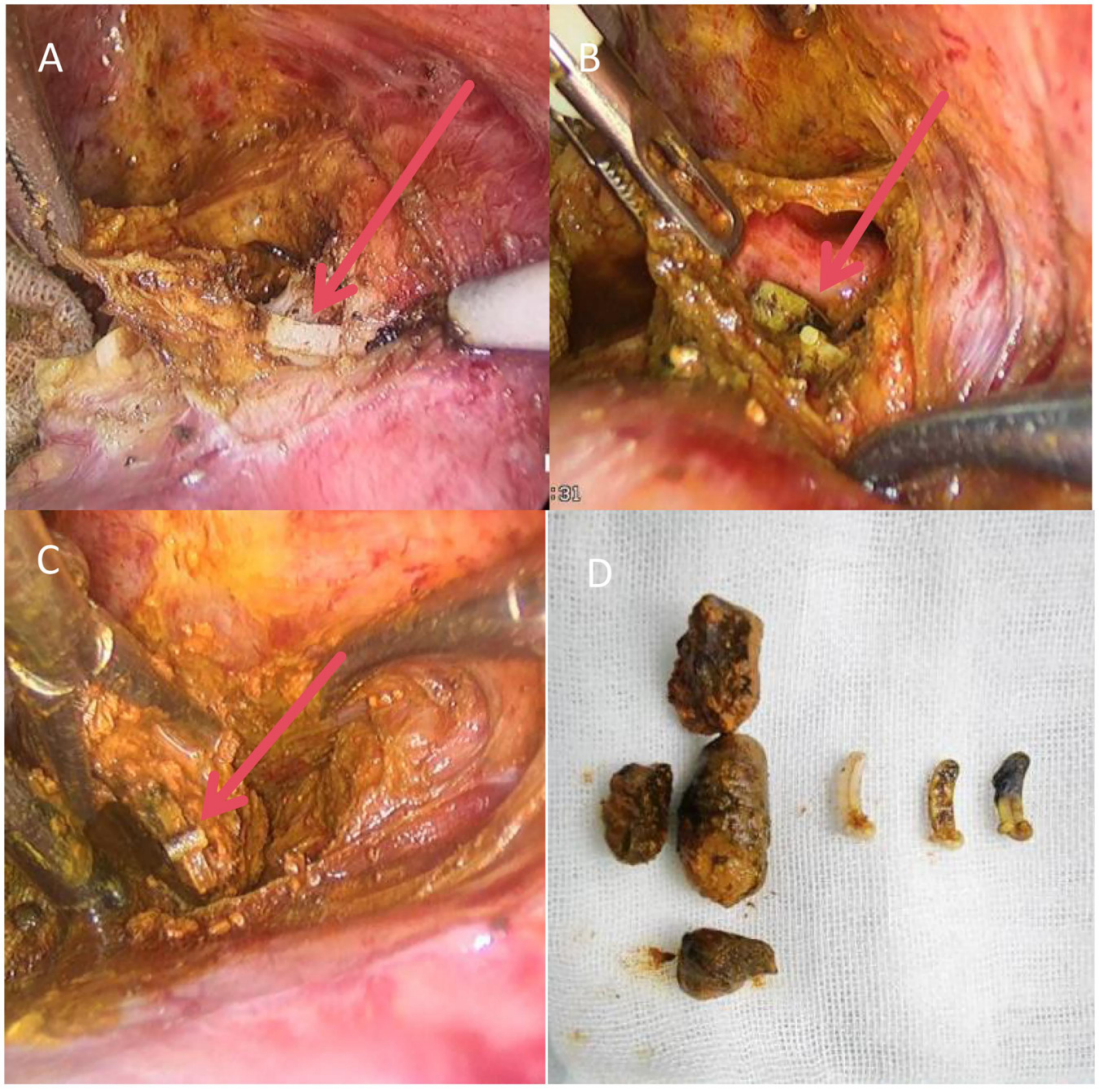
Intraoperative findings of clip locations. **(A)** A Hem-o-lok clip embedded within the muscular layer of the common bile duct wall. **(B)** Two clips that had migrated completely into the lumen, with one situated at the core of a large pigment stone. **(C)** The opened pigment stone, revealing the Hem-o-lok clip at its center, confirming its role as a nidus for stone formation. **(D)** Extracted pigment stones and migrated Hem-o-lok clips on a medical gauze pad.

**Figure 4:**
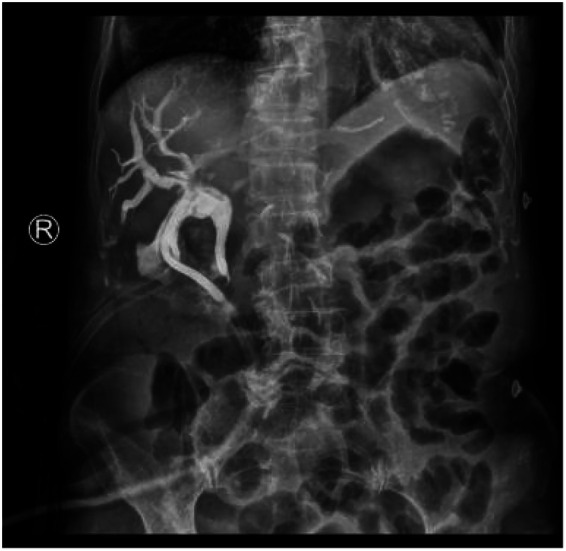
Postoperative T-tube cholangiogram.

Due to the large stone burden extending to the hilum and the clinical severity of cholangitis, endoscopic retrograde cholangiopancreatography (ERCP) was considered high-risk and potentially inadequate. The patient therefore underwent urgent laparoscopic re-exploration. Intraoperative findings were definitive: a total of three Hem-o-lok clips were identified in relation to the common bile duct. One clip was found embedded within the muscular layer of the duct wall (Figure 3A), while two had migrated completely into the lumen. Notably, one of the intraluminal clips was situated at the very core of a large pigment stone (Figures 3B,C), confirming its role as the lead point for stone formation. All clips and stones were successfully extracted via choledochoscopy, and a T-tube was placed for drainage. Bile cultures subsequently grew Enterococcus faecalis, guiding targeted antibiotic therapy with meropenem. The patient's recovery was swift and uncomplicated. Fever and abdominal symptoms resolved promptly, and she was discharged on postoperative day three. Follow-up T-tube cholangiography performed one week later confirmed complete clearance of the biliary tree and free flow of contrast into the duodenum (Figure 4), indicating a successful anatomical and functional outcome.

## Discussion

Laparoscopic cholecystectomy (LC) has been rapidly adopted by surgeons and widely accepted by patients due to its advantages, including shorter hospital stays, fewer complications, and smaller incisions (4). Today, it has become the gold standard for treating benign gallbladder diseases. Although the procedure is generally considered safe and reliable, postoperative complications are not uncommon and can impose additional economic burdens on patients. The most common complications of laparoscopic cholecystectomy include bile duct injury (0.08%–0.5%), bile leakage (0.42%–1.1%), retained common bile duct stones (0.8%–5.7%), postcholecystectomy syndrome (10%–15%), and postcholecystectomy diarrhea (5%–12%) (5). Among these, clip migration is a relatively rare but insidious complication. Its potential to act as a nidus for recurrent biliary stone formation and subsequent cholangitis warrants heightened clinical vigilance (6). We have compiled and reviewed relevant published cases, as detailed in Table 1, which underscore the heterogeneity and diagnostic challenge of this condition. As many scholars note, the early symptoms of clip migration, such as mild abdominal pain and fever, are highly similar to those of common bile duct stones or cholangitis, making it easy to misdiagnose as “post-cholecystectomy syndrome” or simple stone recurrence (7, 8). The spectrum of presentation is broad, ranging from acute episodes as reported by Jiang et al. (11) and Roh et al. (6), to chronic intermittent pain described by Qu et al. (13) and Tan et al. (16). Indeed, some patients remain entirely asymptomatic, with the condition discovered incidentally during imaging follow-ups, a phenomenon documented in the series by Kihara et al. (9) and in individual cases by Pang et al. (10) and Huang et al. (8). In the present case, the patient's initial non-specific manifestations of high fever and chills were initially attributed to a respiratory infection. However, concurrent elevated bilirubin levels raised our suspicion for a biliary source, prompting targeted imaging that ultimately confirmed the diagnosis.

**Table 1:** Clinical characteristics of published cases of clip migration.

First author	Patient age	Symptoms	Signs	Initial surgery	Clip location	Number of clips	Treatment
Jiang (11)	67	Acute RUQ pain	Abdominal tenderness, mild jaundice, fever	LCBDE + T-tube drainage	Common Bile Duct	1	Repeat LCBDE + primary suture
Tan (16)	72	Recurrent RUQ pain	RUQ tenderness	LC + LCBDE	Common Hepatic Duct	4	Laparoscopic bile duct exploration + T-tube drainage
Kihara (9)	81/80/63/74	Case 1: Epigastric pain; Cases 2–4: Asymptomatic	No significant signs	Case1–3: Laparoscopic partial hepatectomy Case 4: LC	Case 1: Duodenal bulb; Cases 2–4: CBD	Case 1: 3; Case 2: 1; Case 3: 1; Case 4: 2	Case 1: Passed spontaneously; Case 2: Choledochotomy; Cases 3–4: ERCP retrieval
Jun Roh (6)	65	Epigastric pain,fever	Scleral icterus,RUQ tenderness	LC	Common Bile Duct	1	ERCP + balloon extraction
Pang (10)	31–83	3 cases asymptomatic; 3 cases RUQ pain	No significant signs	LCBDE/LC	3 cases CBD; 3 cases T-tube sinus	Not specified	Choledochoscopy (3 cases),Roux-en-Y (1 case),bile duct exploration (1 case),PTBD (1 case)
Sun Rou (7)	53	Abdominal pain	RUQ tenderness	LC	Common Bile Duct	1	ERCP + sphincterotomy retrieval
Qu (13)	54	Intermittent epigastric pain	RUQ tenderness	LC + LCBDE + primary suture	Common Bile Duct	2	ERCP + balloon dilation retrieval
Watanabe (12)	73	Fever,malaise	Epigastric tenderness,jaundice	Lap liver S4a + 5 segmentectomy + cholecystectomy	Common Bile Duct	1	ERCP retrieval
Huang (8)	68/74	Case 1: Abdominal distension; Case 2: Asymptomatic	No significant signs	LC + LCBDE + T-tube drainage	Case 1: CBD; Case 2: T-tube sinus	Case 1: 2; Case 2: 1	Case 1: ERCP stone removal; Case 2: Choledochoscopic retrieval
Liu (17)	72	Abdominal pain	Abdominal tenderness	LC	Duodenal bulb	1	Endoscopic retrieval (biopsy forceps)
Wu (14)	62–88	Epigastric pain,fever,chills	RUQ tenderness,jaundice	LC + LCBDE	Common Bile Duct	1–2 per case	Choledochotomy & stone xtraction
Liu (15)	59	Fever,RUQ pain	RUQ tenderness,fever	LC + LCBDE	Common Bile Duct	2	ERCP + basket retrieval

LC, laparoscopic cholecystectomy; LCBDE, laparoscopic common bile duct exploration; ERCP, endoscopic retrograde cholangio-pancreatography; PTBD, percutaneous transhepatic biliary drainage.

These non-specific manifestations necessitate that clinicians maintain a high index of suspicion in patients with a history of biliary surgery, particularly when symptoms recur or when imaging reveals atypical linear high-density shadows within the bile ducts. In our patient, such an imaging finding was present on admission but was initially interpreted merely as part of the stone formation. Previous case reports emphasize that while CT or MRCP may reveal these characteristic high-density shadows, they can be difficult to distinguish from conventional calculi, especially if the possibility of a foreign body is not actively considered (11, 12). The imaging clue of a linear density within a stone, as seen in our case and suggested by others, is pivotal (6). Therefore, a thorough review of imaging for subtle linear or angular metallic densities, particularly within the center of a stone, is crucial for diagnosing clip migration.

Endoscopic Retrograde Cholangiopancreatography (ERCP) is rightly advocated as a first-line, minimally invasive approach for managing migrated clips, with reported high success rates for retrieval (6, 13). This approach has been effectively employed in numerous cases, such as those by Roh et al. (6), Sun Rou et al. (7), Qu et al. (13), Watanabe et al. (12), and Liu et al. (15). However, our decision to proceed directly with laparoscopic common bile duct exploration (LCBDE) in this case was based on a nuanced, multi-factorial assessment. First, the anatomical complexity and stone burden were prohibitive: the common bile duct was packed with stones extending to the hilar region. ERCP in such settings can be technically challenging, associated with prolonged procedure time, an increased need for multiple sessions, and a higher risk of complications such as basket impaction or incomplete clearance (14). Second, the presence of acute suppurative cholangitis mandated definitive and efficient source control. While ERCP with sphincterotomy provides drainage, surgical exploration allows for direct visualization, complete removal of all stones and the embedded foreign body nidus under choledochoscopic guidance, and establishment of secure T-tube drainage—a strategy particularly advantageous in the setting of active infection (15). This stratified approach aligns with the management of other complex cases in the literature. For instance, Jiang et al. (11), Tan et al. (16), and Wu et al. (14) also utilized surgical exploration (LCBDE or choledochotomy) in their patients, often in scenarios involving multiple clips, large stone burdens, or complex anatomy. As summarized in Table 1, this tailored strategy exemplifies the principle of individualized management: endoscopic retrieval is preferred for accessible, single clips, whereas surgical exploration is reserved for more complex presentations (6, 13, 16).

The mechanism of clip migration is multifactorial, resulting from the interplay of surgical technique and subsequent biological responses. As noted by Jiang and Wu, local inflammation (e.g., from bile leakage or cholangitis) weakens peri-ductal tissues, creating a pathway for clip erosion (11, 17). This process can be conceptualized as a chronic foreign body reaction. The initial clip placement, if too close to the common bile duct or on inflamed tissue, provides the mechanical premise (13). Subsequently, a persistent inflammatory milieu, characterized by the release of proteolytic enzymes and cytokines, facilitates tissue necrosis and remodeling around the clip (18). This biological erosion, compounded by mechanical stresses from peristalsis or adjacent structures over time, can ultimately lead to transmural migration into the duct lumen. The finding in our case of one clip embedded within the muscular layer of the duct wall (Figure 3A) visually corroborates this transitional stage of erosion. Additional pathways include migration along a T-tube sinus tract, as proposed by Pang et al. (10) and illustrated in cases by Huang et al. (8) and Pang et al. (10), or migration to unusual sites like the duodenal bulb via adhesions, as reported by Liu et al. (17). The series by Kihara et al. also demonstrates that clips can migrate far from their original site, even into the duodenum (19). Our patient's history of suppurative cholecystitis during the initial surgery and T-tube placement likely contributed to such a pro-inflammatory and mechanically altered local environment, underscoring the importance of meticulous technique and postoperative inflammation control in prevention.

A critical component often underreported in rare complications is substantive long-term follow-up, which is essential to validate therapeutic success and assess definitive prognosis (20, 21). In this case, the patient's management extended beyond the immediate postoperative period. Systematic follow-up confirmed a durable positive outcome: a T-tube cholangiogram at one week post-operation demonstrated a patent and clear biliary tree (Figure 4). The T-tube was successfully removed after six weeks following confirmation of distal bile duct patency. Subsequent clinical and ultrasonographic evaluations at one, three, and six months revealed the patient remained asymptomatic, with normal liver function tests and no evidence of recurrent stones or ductal dilation. This documented, event-free follow-up period of over six months provides crucial closure to the case. It strongly indicates that the chosen surgical intervention not only resolved the acute episode of cholangitis but also eliminated the underlying nidus, thereby restoring normal biliary physiology and minimizing the risk of recurrence.

Although some scholars suggest reducing the number of clips, using absorbable clips, or employing ultrasonic scalpels to mitigate migration risks, reports indicate that absorbable clips can also migrate and serve as stone nidi (10, 13). Clip-free techniques, while promising, face limitations in cost and technical requirements that hinder widespread adoption. Therefore, fundamental prevention should emphasize standardized surgical techniques. As emphasized by Wu et al., ensuring clips are placed at a safe distance (at least 0.5–1.0 cm) from the common bile duct, avoiding application on frankly inflamed or necrotic tissue, and maintaining enhanced postoperative follow-up for high-risk patients currently represent the most feasible and evidence-based preventive strategies (17–21).

## Conclusion

In summary, this case highlights Hem-o-lok clip migration as a consequential, albeit rare, long-term complication of laparoscopic biliary surgery. The insidious nature of this condition, with its non-specific symptoms often masquerading as common bile duct stones or cholangitis, necessitates a high index of suspicion in patients presenting with late-onset biliary symptoms after cholecystectomy. Diagnosis relies critically on advanced imaging, with a careful search for linear hyperdensities within stones being a key radiological clue. The pathogenesis involves a multifactorial interplay, where initial technical factors—such as clip placement on inflamed tissue—combine with a chronic, biologically-driven inflammatory response to facilitate gradual erosion and migration into the bile duct lumen. This understanding underscores the importance of meticulous surgical technique during the index operation. Our management strategy, opting for laparoscopic common bile duct exploration over first-line ERCP, was guided by the specific clinical context of a large stone burden extending to the hilum and concurrent acute suppurative cholangitis. This decision aligns with a stratified therapeutic approach that prioritizes definitive source control and complete clearance in complex scenarios. The patient's excellent recovery and sustained well-being over six months of follow-up confirm the efficacy and durability of this intervention. Ultimately, this report emphasizes that vigilance for clip migration should extend into the long-term postoperative period. Enhancing surgical precision during clip application and maintaining awareness of this potential complication are paramount to its prevention and timely management, thereby safeguarding long-term patient outcomes.

## Data Availability

The original contributions presented in the study are included in the article/Supplementary Material, further inquiries can be directed to the corresponding author.
